# The Disturbed Microbial Niches of Itchy Scalp

**DOI:** 10.1111/jocd.70010

**Published:** 2025-01-23

**Authors:** Xuejing Li, Fang Yang, Yingxin Ma, Mengmeng Zhang, Yan Zhang, Menghui Zhang

**Affiliations:** ^1^ State Key Laboratory of Microbial Metabolism, Joint International Research Laboratory of Metabolic and Developmental Sciences, School of Life Sciences and Biotechnology Shanghai Jiao Tong University Shanghai China; ^2^ Henkel (China) Investment Co. Ltd., R&D Shanghai China; ^3^ Key Laboratory of Systems Biomedicine (Ministry of Education), Shanghai Center for Systems Biomedicine Shanghai Jiao Tong University Shanghai China

**Keywords:** *Cutibacterium*, microbial disequilibrium, microbial niche, non‐resident microbes, scalp pruritus, *staphylococcus*

## Abstract

**Background:**

Scalp itch without evident cause is an uncomfortable symptom that annoys many people in life but lacks adequate attention in academic.

**Aims:**

To investigate the relationship between scalp itching and microorganisms, and identify the key microbes and predicted functions associated with scalp itching, furtherly to provide useful targets for scalp itch solution.

**Methods:**

We performed microbial comparison between 44 normal subjects and 89 subjects having scalp itching problem with un‐identified origin based on 16S rRNA gene sequencing and ddPCR (digital droplet PCR), and identified itch relevant microbes and predicted functions. To minimize false positive findings, another 43 itchy subjects were used for independent validation.

**Results:**

The three most abundant bacterial genus *Cutibacterium*, *Lawsonella, Staphylococcus* and predominant fungi *Malassezia* were not significantly different between the normal and itchy subjects, but an imbalance between *Cutibacterium* and *Staphylococcus* occurred in itchy scalp. As the predominant function contributor in the community, various *Cutibacterium* ASVs (amplicon sequence variants) were detected in itchy or normal subjects with low abundance and were subject specific. Some non‐resident microbes from environment such as *Leptolyngbya ANT.L52.2* and *Pseudomonas* were enhanced in itchy scalp and occupied an important ecological niche. The severity of itch was alleviated when these low abundant and low subject coverage ASVs were reduced or diminished.

**Conclusions:**

Our findings raise the attention to (1) the ratio of *Cutibacterium* to *Staphylococcus* and (2) the low abundant bacteria on scalp, and provide potential solutions to ease scalp itch.

**Abbreviations**
ANOVA: analysis of variance
ASFS: adherent scalp flaking score
ASVs: amplicon sequence variants
ddPCR: digital droplet PCR
KEGG: Kyoto Encyclopedia of Genes and Genomes
MANOVA: multivariate analysis of variance
PCR: polymerase chain reaction
PICRUSt2: phylogenetic investigation of communities by reconstruction of unobserved states 2
ROBPCA: robust principal component analysis
rRNA: ribosomal RNA
VAS: visual analogue scale

## 1 Introduction

Scalp pruritus is a frequent and distressing problem affecting approximately 13%–45% of the population in the world [1, 2]. Identifying the origin of pruritus is crucial for a precision diagnosis and treatment. Many factors can induce scalp pruritus, some cases of which are commonly caused by various underlying diseases, such as seborrheic dermatitis and psoriasis, but others appear without obvious diagnosis [3, 4]. A challenge in clinical practice is that there are considerable people suffered from this annoyed symptom without evident cause and currently there lacks effective therapy or satisfied daily chemical products for this type of patients [4]. Up to date, researches on scalp pruritus without determined origin are quite limited.

Microbial niche refers to the distribution and performance of microorganisms in a space [5]. The change of the microbial niche in a community could result in compositional and functional dysbiosis [5]. Studies on some scalp diseases, for instance, dandruff, seborrheic dermatitis, and alopecia areata had reported disturbed microbial niches [6, 7, 8]. The related findings especially focused on the predominant fungal and bacterial genera—*Malassezia*, and *Cutibacterium* and *Staphylococcus*, respectively [6, 9, 10, 11].

However, in the scalp region that one of the anatomical areas frequently manifested with pruritus, few studies have investigated the role of scalp microbiota in itch. Our preliminary study on scalp itchy subjects indicated that the scalp bacterial composition was of large variation among individuals and was different between the itching and non‐itching sites, suggesting that there might be a certain relationship between scalp itching, and microbiota [12]. This observation encouraged us to gain deeper understanding the impact of scalp microbiota on itching.

In this study, we first obtained the scalp microbial information of 89 itchy and 44 non‐itchy subjects using relative quantification by 16S rRNA gene sequencing and absolute quantification by ddPCR. Then the differential microbial compositions and predicted functions between the two subject groups were identified through bioinformatic analysis and multivariate modeling. Finally, a cleaning detergent was applied to another 43 itchy subjects for 28 days to make microbial fluctuant and to change itchy severity with the purpose to verify important clues relevant to scalp itch. Our findings provide useful targets for scalp itch solution.

## 2 Materials and Methods

### 2.1 Volunteer Recruitment and Group Allocation Design

The volunteer recruitment was performed at Shanghai China‐norm Quality Technical Service Co. Ltd. (China‐norm, Shanghai, China), a third‐party testing organization. Briefly, 323 apparently healthy female subjects aged between 18 and 60 years were randomly selected from the local volunteer database. After an initial questionnaire, the subjects were invited to the China‐norm and further recruited under the evaluation of the same professional dermatologist according to pre‐defined criteria: (1) had visual analogue scale (VAS) ≥ 6 or ≤ 2 [13]; (2) had adherent scalp flaking score (ASFS) ≤ 4 in any of the six pre‐defined scalp regions [14]; (3) were not during pregnant and lactation period, and had no pregnant willing during this test; (4) had no oral and external antibiotics, immunosuppressant, chronic anti‐inflammatory and anti‐histamine drugs taken within 3 months; (5) had no shampoos and/or therapeutic products applied to the scalp which declare to have anti‐dandruff, anti‐psoriatic or anti‐seborrheic dermatitis functions within 1 month; (6) had no hair perming and/or coloring within 1 month; (7) had no concomitant scalp diseases (like seborrheic dermatitis and psoriasis), diabetes or other chronic diseases. As a result, 176 subjects finally remained that 132 of them had itchy scalp with VAS higher than 6 and 44 were normal with VAS lower than 2 (Details see Figure S1). All the subjects were provided written informed consent willing to participate in complete test process and had been explained the procedure and the purpose of the study.

To identify and validate the difference between the itchy and normal subjects, the 132 itchy subjects were then randomly separated into training group (*n* = 89) and validation group (*n* = 43). The different/discriminant analysis was always performed with the training group and the normal group (*n* = 44), and the validation group was only used as an independent test set to validate the differences. All the groups simultaneously received the microbial sampling and physiological parameter measurements on Day 0. In addition, the validation group had a 28 days' intervention (wash scalp every 2 days) with a pure detergent. The pure detergent is a basic shampoo formula with water, surfactants (mainly cocamidopropyl betaine, N‐(coconut oil acyl)‐N‐methyl taurine, sodium salt, coco‐glucoside, and sodium C14‐16 olefin sulfonate), humectant (glycerin), hair conditioning agent and preservative (Na‐benzoate) but without any actives so that it can help clean scalp gently but has no targeted bioactivity to kill or protect microbes. Unless otherwise stated, the methods for data collection followed the same procedures and the microbial measurement was performed on the same sequencing run.

### 2.2 Scalp Microbial Sampling and Physiological Parameter Measurements

The recruited subjects were informed not to wash scalp for 2 days prior to the sampling procedure. On the day for microbial sample collection, each subject stayed under a controlled environment with relative humidity of 50% ± 5% and temperature at 21°C ± 1°C for 30 min. Subjects firstly finished VAS scoring and 3S questionnaire [15], and were examined by a dermatologist to get dandruff grading according to the adherent scalp flaking scale (ASFS). Then, the scalp sampling region(s) were evaluated and marked by the dermatologist in terms of scalp condition. Each subject with normal scalp had one region of the non‐itchy site marked, while subject with itchy scalp had two regions marked, one was the itchy site pointed out by the subject and the other was the non‐itchy site on the symmetric region of the scalp. Finally, the microbial sampling of the marked sites was performed in a UV sterilized room using previously described procedure with minor modification [12]. Briefly, five swabs were used in each microbial sampling region (0.5 × 6 cm^2^ in area) surrounding a marked site. The swabs were immediately put in sterilized tubes which were frozen in dry ice and then stored at −80°C until DNA extraction.

After microbial sampling, the scalp hydration and pH value closely near the marked sites were measured with Dermalab Combo skin tester (Cortex, Denmark), the sebum content was obtained with Meibometer MB560 (Courage‐Khazaka, Germany) and the erythema index was measured with Tri‐spectrum scalp detector in polarized light mode and calculated with software Image Pro (Media Cybernetics, US).

### 2.3 DNA Extraction, 16S rRNA Gene Sequencing, and ddPCR Quantification of Microbial Taxa

The scalp microbial DNA was extracted from the swabs using the DNeasy Blood & Tissue Kit (Qiagen, ID: 69506) according to the manufacturer's protocol with minor modification, and then were put into two tubes for sequencing and quantification respectively, being stored at −80°C until use [12].

The 16S rRNA gene sequencing was performed at Shanghai Personalbio Technology Co. Ltd. (Shanghai, China). Briefly, the extracted DNA was amplified with the 338F (5′‐ACTCCTACGGGAGGCAGCA‐3′) and 806R (5′‐GGACTACHVGGGTWTCTAAT‐3′) primers specific for the V3‐V4 hypervariable regions. The sequencing was processed on the Illumina NovaSeq platform and 250 bp paired‐end reads were generated.

The microbial quantification was performed using Bio‐Rad QX200 ddPCR system (Bio‐Rad Laboratories, USA) according to the standard operating protocol. The primers used for amplification are shown in Table S1, and the amplification procedure was as follows: 95°C for 10 min, and 40 cycles of 95°C for 30 s and 57°C for 60 s, 90°C for 2 min, 4°C hold. The quantitative data was obtained by QX200 Droplet Reader (Bio‐Rad) and QuantaSoft Software (Bio‐Rad).

### 2.4 Bioinformatics and Statistical Analysis

The adaptor and primers of the raw 16S rRNA gene sequencing data were trimmed off, and the paired‐end reads were assembled according to at least 12 bp overlaps by USEARCH (version 11.0667). The assembled reads were then put in QIIME2 (version 2021.4, https://qiime2.org/) for quality control, chimera removal and ASVs assignment by DADA2 with minor modification using in house Python script [16]. After the calculation of the ASV abundance matrix, normalization was done by downsizing ASV reads to 25 000 per sample to reduce the influence of sequencing depth. The representative sequence of each ASV was aligned to SILVA v138 database to get taxonomic information.

Shannon index, the observed number of ASVs, the Pielou evenness and Bray Curtis distance were calculated with QIIME2. MANOVA (Multivariate analysis of variance) and ANOVA analysis (Analysis of Variance) were done under Matlab environment (2019b; The MathWorks, Natick, MA, USA). ROBPCA (Robust principal component analysis) was used for detecting and removing outliers [17]. The Random Forest Modeling was performed with R packaging (version 4.0.2, http://ww.r‐project.org/). The Spearman correlation coefficients were calculated in IBM SPSS Statistics software (SPSS version 19; IBM Corp). The metabolic and functional activities of the bacterial communities were predicted with PICRUSt2 (Phylogenetic Investigation of Communities by Reconstruction of Unobserved States 2) using KEGG (Kyoto Encyclopedia of Genes and Genomes) pathways (https://www.kegg.jp).

The statistical analysis was done by GraphPad Prism (version 8.02; San Diego, CA, USA) and R package (version 4.0.2, http://ww.r‐project.org/). The differences between two groups were evaluated by parametric *t*‐test and nonparametric test as appropriate according to normality. *p*‐value less than 0.05 was considered statistically significant unless otherwise specified.

## 3 Results

### 3.1 Demographic and Physiological Characteristics of the Normal and the Itchy Scalps

We compared the demographic and physiological characteristics of the itchy training group (Itch) and the normal group (Normal) (Table 1). The VAS of the Itch group was 7.61 ± 0.77, significantly higher than that of the Normal group (0.48 ± 0.45). Of note, the higher VAS scores of the itchy group were not due to the dandruff, as we only included subjects with low ASFS values in this study and thus the two groups had similar ASFS scores. Corresponding to the higher VAS values, the itchy subjects also had significantly higher 3S scores which were relevant to scalp sensitivity. Though we did not observe significant differences in sebum content and pH value between the two groups, we found that the Itch group had significantly higher stratum corneum hydration level, suggesting a higher degree of hydration might be relevant to scalp itch. The Itch group also had higher erythema index, a measure of the severity of scalp redness, though this trend was not significant.

**TABLE 1:** Physiological indexes of subjects.

Subject group	Normal (*n* = 44)	Itch (*n* = 89)	*p*
VAS	0.48 ± 0.45	7.61 ± 0.77	< 0.0001
3S scores	0.57 ± 0.69	2.37 ± 1.25	< 0.0001
ASFS	2.94 ± 2.79	3.40 ± 2.35	0.1282
Hydration (μS)	27.91 ± 14.06	55.17 ± 44.57	< 0.0001
Sebum content (a.u.)	337.59 ± 193.40	341.38 ± 161.05	0.8646
pH value	4.76 ± 0.61	4.59 ± 0.44	0.1162
Erythema index (a.u.)	0.06 ± 0.06	0.07 ± 0.07	0.2726

*Note:* Values were shown as Mean ± SD. The *p*‐values were calculated by Mann–Whitney test.

### 3.2 Microbial Diversity and Composition of the Normal and Itchy Scalps

After DNA extraction, 16S rRNA sequencing, sequence data pre‐processing and outlier removal, we obtained high‐quality reads from the 83 itchy subjects and the 41 normal subjects. After the normalization for sequencing depth, we obtained 7786 ASVs that belong to 1113 genera in total.

**FIGURE 1:**
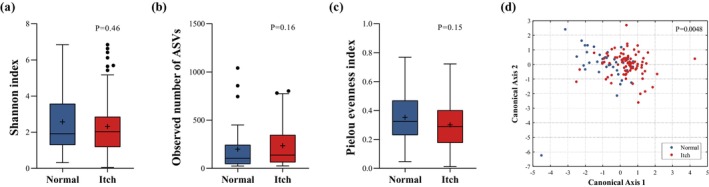
The scalp alpha‐ and beta‐ bacterial diversity of the normal (Normal) and the itchy (Itch) subjects. Alpha‐diversity was evaluated using (a) Shannon index, (b) the observed number of ASVs and (c) Pielou evenness index. The observed number of ASVs and Pielou evenness index were used to indicate the microbial richness and evenness respectively, and Shannon index took both the richness and evenness into account. The higher value of Shannon index, the higher richness and evenness of the microorganisms. (d) The multivariate analysis of variance (MANOVA) plot of scalp microbiota based on the Bray Curtis distance. Bray Curtis dissimilarity was used to assess beta diversity of bacterial community structure.

The results from the observed ASVs and evenness indexes of the microbiota data indicated that the itchy subjects had higher microbial richness but less microbial evenness. Though these three alpha diversity differences between the Normal and the Itch groups were not significant (Figure 1a–c), we observed significant microbial compositional differences (*p* = 0.0048) (Figure 1d). Our previous study restricted on itchy subjects had shown that the microbiota on the itchy site was significantly different from that on the non‐itchy site [12]. In this study we also sampled the non‐itchy site of the itchy subjects. We found that the difference between the itchy and non‐itchy sites within the itchy subjects was far less than the difference between the itchy subjects and the normal subjects (Figure S2). This comparative result suggested that for the itchy subjects, the microbial composition was overall changed no matter in itchy or non‐itchy region.

**FIGURE 2:**
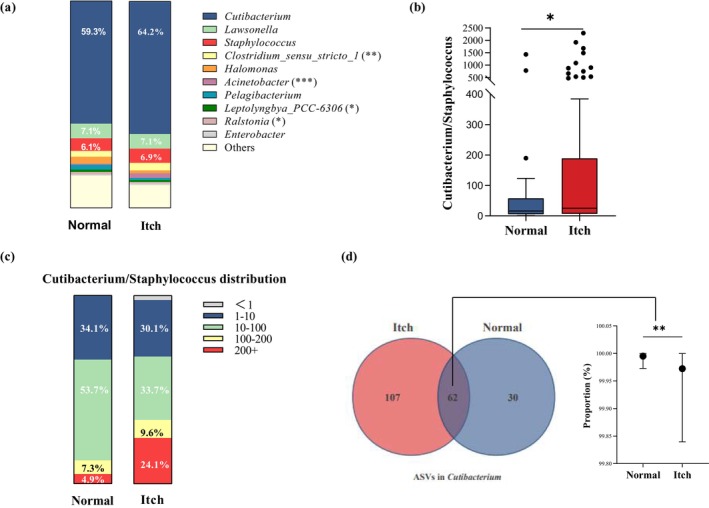
The comparison of predominant scalp bacterial genera between the normal (Normal) and the itchy (Itch) subjects. (a) The top 10 dominant bacterial genera. (b) The ratio of *Cutibacterium* to *Staphylococcus*. The *p*‐value was calculated by *t*‐test with Welch's correction; (c) the distribution of the *Cutibacterium* genus/*Staphylococcus* genus values. (d) The venn diagram of *Cutibacterium* ASVs and the proportion in abundance of the common *Cutibacterium* ASVs to all *Cutibacterium* ASVs. The *p*‐values were calculated by Mann–Whitney test. *p < 0.05, **p < 0.01, ***p < 0.001.

At the genus level, *Cutibacterium*, *Lawsonella*, *Staphylococcus*, *Clostridium sensu stricto* 1, *Halomonas*, *Acinetobacter*, *Pelagibacterium*, *Leptolyngbya PCC‐6306*, *Ralstonia*, and *Enterobacter* were the 10 most abundant bacterial genera in the itchy scalp (Figure 2a). Whether in normal and itchy subjects, the most abundant genera observed across all samples were *Cutibacterium*, *Lawsonella* and *Staphylococcus*, which accounted for more than 70% of the total bacteria. These three genera were not significant different between the two groups. The genera *Clostridium sensu stricto* 1, *Acinetobacter*, *Leptolyngbya PCC‐6306*, *Ralstonia*, and *Enterobacter* were also detected at both normal and itchy scalp sites. Among them, *Ralstonia* was significantly more abundant in normal scalp, while *Clostridium sensu stricto* 1, *Acinetobacter*, and *Leptolyngbya PCC‐6306* were more abundant in itchy scalp significantly.

Though the contents of *Cutibacterium* and *Staphylococcus* were similar in the two groups, the Itch group had significant higher ratio of *Cutibacterium* to *Staphylococcus* than that of the Normal group (Figure 2b). The Normal group had 87.8% subjects with this ratio between 1 and 100 while the Itch group had 63.8% (Figure 2c). In particular, 24.1% subjects in the Itch group had this ratio higher than 200 or even more than 500 (Figure 2b,c), suggesting that the balance between these two dominant microbes are important to the scalp itchy status.

The Normal group and the Itch group had 62 ASVs of *Cutibacterium* in common (details see Table S2). These shared ASVs had absolute predominance in both groups and the sum of their abundances was significantly higher in the Normal group (Figure 2d). Among them, ASV1 and ASV4 were detected in more than 90% of all the subjects and the averaged sum of these two ASVs accounted for about 60% of the total ASV abundances (Table S2). For the rest common ASVs, most of them were of low sample coverage, that is, were detected in less than 50% of the subjects. Furthermore, the Itch group had 107 unique ASVs of *Cutibacterium*, far more than 30 in the Normal group. However, no matter in the Itch or Normal groups, these 137 unique *Cutibacterium* were all with very low sample coverage (mostly appeared in one or several subjects) and in low abundance.

### 3.3 Quantification of *Cutibacterium*, *Staphylococcus*, and *Malassezia* by ddPCR

The quantities of the dominant bacteria had been considered as important factors causing some scalp disorders, such as dandruff and seborrheic dermatitis [9, 18]. However, 16S rRNA gene sequencing only provided relative abundance of microbes in each sample. Meanwhile, since *Malassezia* is also an important microbe to scalp status, in order to get more quantitative measurement of the dominant microbes, we further performed quantitative analysis on the *Cutibacterium*, *Staphylococcus*, and *Malassezia* of 30 normal samples and 62 itchy sample which were qualified for ddPCR. In contrast with the relatively lower *Cutibacterium* and *Staphylococcus* abundances in the Normal group revealed by 16S rRNA gene sequencing, the ddPCR showed these two genera were in higher quantities in the Normal group (Figure S3a,b). Consistent with the result obtained by 16S rRNA gene sequencing, the quantity ratio of *Cutibacterium* to *Staphylococcus also* showed a higher trend in Itch group (Figure S3d). In addition, the quantities of *Malassezia* did not differ in two groups (Figure S3c).

### 3.4 The Key Bacteria Relevant to the Itchy Scalp

**FIGURE 3:**
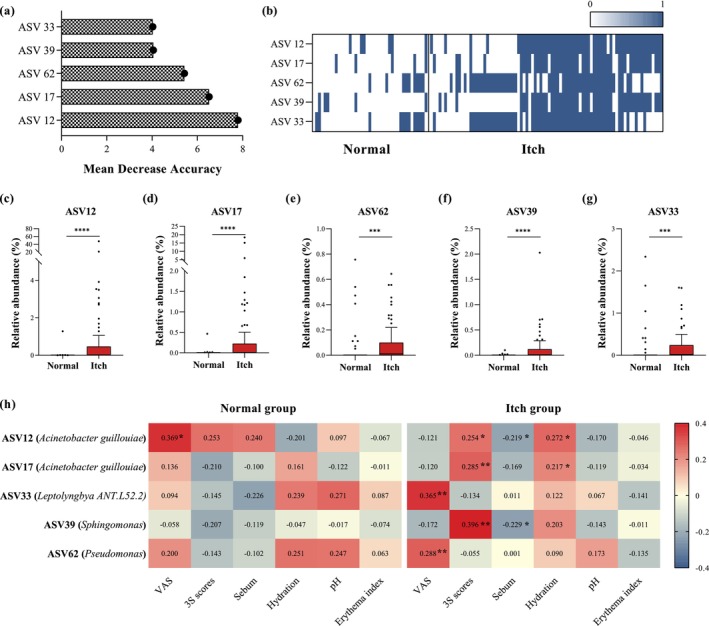
Identification scalp itch relevant ASVs by random forest analysis. (a) Mean decrease accuracy (MDA) assigned from ASVs. Displayed cutoffs are MDA > 4.0 and sample coverage> 20%. The higher value of MDA, the more important of the corresponding ASV. (b) The distribution heatmap of the differential ASVs in normal and itch scalp. One means present, 0 means non‐present. The relative abundance of (c) ASV12 ( *Acinetobacter guillouiae* ), (d) ASV17 ( *Acinetobacter guillouiae* ), (e) ASV62 (*Pseudomonas*), (f) ASV39 (*Sphingomonas*), and (g) ASV33 (*Leptolyngbya ANT.L52.2*) in normal and itchy scalp. (h) The correlation heatmap between the five differential AVSs and phenotypic measurements within each group. *p＜0.05, **p＜0.01, ***p＜0.001, ****p＜0.0001.

We conducted Random Forest analysis to further identify the bacteria that might be relevant to scalp itch. At the ASV level, with the criteria MDA (Mean decrease accuracy) > 4.0 and sample coverage > 20%, we filter out five ASVs significantly enriched in the Itch group, including ASV12 and ASV17 ( *Acinetobacter guillouiae* ), ASV 62 (*Pseudomonas*), ASV39 (*Sphingomonas*), and ASV33 (*Leptolyngbya ANT.L52.2*) (Figure 3). A literature search showed that all these ASVs are environmental bacteria and are non‐resident scalp bacteria. But in our case, they occupied an important ecological niche of the itchy scalp. The correlation analysis (Figure 3h) illustrates the relationships between key ASVs and demographic variables for each group: normal and itch group. Compared with the normal group, the more significant correlation was observed in itch group. In the itch group, ASV33, and ASV62 showed a significantly positive correlation with itchy severity. ASV12 and ASV17 correlated with 3S scores and scalp hydration positively, and with sebum negatively. Additionally, there were correlations between ASV39 and 3S scores (*r* = 0.396) and ASV39 and sebum (*r* = −0.229).

### 3.5 The Key Functional Pathways Related to the Itchy Scalp

In the normal or itchy scalp, the main bacterial metabolic activities predicted by PICRUSt2 included the biosynthesis of ansamycin, amino acid metabolism, metabolism of cofactors and vitamins, and carbohydrate metabolism (Figure S4a). Among these, the most abundant pathway was biosynthesis of ansamycin and it was mainly contributed by *Cutibacterium*. This might be an important reason for the dominant ecological niche of *Cutibacterium* on the scalp due to the antibacterial activity from ansamycin. In addition, *Cutibacterium* was also the main contributor for D‐Glutamine and D‐glutamate metabolism, D‐Alanine metabolism, Lipoic acid metabolism, Thiamine metabolism, and biotin metabolism.

We further obtained 18 key pathways through ANOVA analysis (Figure S4b). For the 16 pathways enriched in the normal scalp, the pathways of Propanoate metabolism, Streptomycin biosynthesis, Phenylalanine metabolism and ABC transporters were mainly contributed by the predominant bacteria. The rest two (Rentinol metabolism, Butirosin, and neomycin biosynthesis) enriched in the itchy scalp and their contributor were mainly non‐resident scalp bacteria, suggesting they occupied an important metabolic niche in the itchy scalp.

### 3.6 Hypothesis for Scalp Itch and the Validation

Taking above results together, we hypothesis that the occupation of predominant bacteria and non‐resident bacteria on the scalp both play important roles on the scalp itch. We used the data from the validation group before (D0) and after (D28) a 28 days' intervention to check the consistence of our findings from comparison of the Normal and Itch group. After the intervention by using a pure detergent, 40 out of the 43 subjects of the validation group had finished scalp microbial sampling and physiological parameter measurements at the two time points and 16 subjects had paired microbial sequencing information. All the 40 subjects had improvement in itch severity and dandruff severity in certain degree that the VAS, 3S scores and ASFS were all decreased significantly after intervention (Table S3). Previously we observed that hydration level in the Itch group was significantly higher than that in the Normal group (Table 1). For the validation group, the non‐significant reduction of hydration under the significant improvement of itch severity suggested that hydration level might not be an important index for status of itchy scalp. In contrast with the no difference of sebum content between the Normal and the Itch group, the validation group had sebum content significantly decreased from 335.25 to 265.30. This inconsistence change in the itchy subjects might also suggested that the sebum content might also not an important index for status of itchy scalp. The dropped sebum content might be due to the clean effect of shampoo. Additionally, results from the validation group indicated that pH and Erythema index were also not itchy relevant.

**FIGURE 4:**
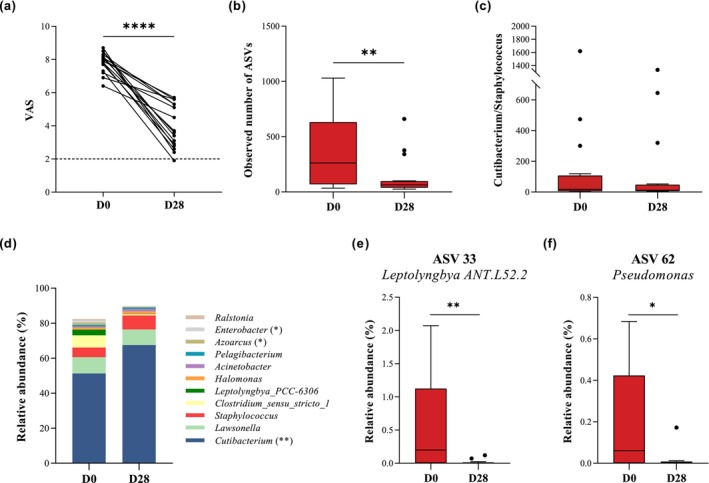
The change of (a) VAS, (b) observed number of ASVs, (c) the ratio of *Cutibacterium* to *Staphylococcus*, (d) the abundance of predominant genus, (e) ASV 33, and (f) ASV 62 after intervention. *p＜0.05, **p＜0.01, ****p＜0.0001..

All the 16 subjects who had complete microbial information in the validation group had alleviated VAS score after detergent intervention, in particular that one person had VAS score reduced to below 2 (Figure 4a). Along with the improvement on itch after intervention, the number of ASVs was significantly reduced (Figure 4b). Considering that the Itch group had higher ASV number than that of the Normal group, we deduced a reduction of ASV diversity might contribute to the alleviation of itchy severity. Both abundances of *Cutibacterium* and *Staphylococcus* displayed an increasing trend with the amelioration of itching, whereas the relative abundance of *Cutibacterium* significantly increased. Of note, the ratio of *Cutibacterium* to *Staphylococcus* also had decreasing trend after the intervention (Figure 4c), implying that this ratio was itch relevant, though there was no significant difference between the two time points. Meanwhile, *Lawsonella*, *Clostridium sensu stricto* 1, *Halomonas*, *Acinetobacter*, *Pelagibacterium*, and *Leptolyngbya PCC‐6306* showed a decreasing trend in certain degree, *Azoarcus*, and *Enterobacter were* decreased significantly (Figure 4d). For the five key ASVs we identified relevant to scalp itch in the previous RF modeling, the abundances of ASV33 (*Leptolyngbya ANT.L52.2*) and ASV62 (*Pseudomonas*) were significantly decreased but the other 3 ASVs were not (Figure 4e,f).

## 4 Discussion

In this study, we systematically investigated the scalp microbial differences between subjects with and without itch problem using relative and absolute quantification technology for microbes. Meanwhile we set an independent itchy group for validation in order to minimize the positive findings related to scalp itch. We found the microbial difference between the normal subjects and itchy subjects was higher than the difference within the itchy group, which indicated that the itchy scalp had significant changes in microbes even in non‐itchy region and should be paid attention. More important, we demonstrated that the balance between *Cutibacterium* and *Staphylococcus* and the localization of non‐resident microbes from environment were potential key factors affecting status of scalp itch.

We found with or without scalp itch, *Cutibacterium*, *Lawsonella* and *Staphylococcus* are predominant bacteria on scalp, suggesting they occupy important ecological niches, which was also reported in many studies [19, 20, 21]. Relative quantification by 16S rRNA gene sequencing and absolute quantification by ddPCR both indicated that these three predominant bacteria and fungi *Malassezia* were not significantly different between the itchy and normal subjects. However, the ratio of *Cutibacterium* to *Staphylococcus* was relevant to scalp itch. Majority normal subjects had this ratio between 1 and 100, whereas the values of many itchy subjects are outside of this region, in most cases were larger than 100, suggesting this ratio might be useful as a scalp itch index. Previous studies reported that *Cutibacterium* and *Staphylococcus* are competitive on the scalp and the imbalance between them was relevant to some scalp disorders such as dandruff [8, 9]. Our study provided new evidence that this imbalance was also related to scalp itch.

We found that the itchy scalp had higher number of ASVs that mainly were in low abundance and were personal dependent. *Cutibacterium* was the most predominant microbe in the scalp and contributed predominant functions. We had detected 199 *Cutibacterium* ASVs in total from the 83 itchy and 41 normal subjects, with 107 uniquely distributed in itchy subjects, suggesting there is necessary to subdivide the role of different *Cutibacterium* to itch/health. Only 62 of the 199 *Cutibacterium* ASVs were shared by the two groups, and only 2 (ASV1 and ASV4) existed in more than 90% of subjects. The low abundant *Cutibacterium* ASVs uniquely localized in few subjects, particularly in itchy subjects, might contribute to scalp itch since removing them with detergent could alleviate itchy severity. Another itch origin might come from some environmental non‐resident bacteria, at least including ASV33 (*Leptolyngbya ANT.L52.2*) and ASV62 (*Pseudomonas*) since we validated that reduction of them indeed could ease itch. Thus, we think lessen low abundant bacteria might be a good method for treating scalp itch though the underlying mechanism caused still warrants further exploration.

Some physiological characteristics displayed inconsistent relationships to the itchy status. The pH, water content and sebum are considered to be important in keeping the conditions of the scalp skin and affecting the balance of the microorganisms [22, 23]. In our study, we did not see the relationship between the pH and scalp itch. Though we observed significant higher level of hydration in the Itch group, this phenomenon was not supported by the validation group. This result could suggest that hydration level is not relevant to scalp itch but it also might be due to the limited sample size and large variation within the subjects. The validation group had significant lower sebum after using the clean detergent that has the ability to reduce sebum. However, the Itch group had non‐significant sebum level compared with the Normal group, which also did not support the relationship between the sebum and itch condition. Hence, we think the influences from the skin hydration, sebum level and pH to the scalp itch need further exploration with specific experiment design and enough samples.

Since this study was limited to Chinese females, our findings might not be generalized to other populations under different conditions such as with different gender, age, locations or races. But these findings could be served as references in broader investigations in the future. Additionally, the deep study at microbial species level will help the exploration of itch mechanisms no matter by using ddPCR or sequencing method, especially in dominant microbes' study (*Malassezia*, *Cutibacterium* and *Staphylococcus*). In terms of clinical application, we think (1) the ratio of *Cutibacterium* to *Staphylococcus* and (2) the low abundant bacteria on scalp provide potential solutions to ease scalp itch. In the validation group, the pure detergent combined with hair washing regularly could help release itch. We observed the itch relief accompanied by the decrease of microbial richness and low abundant bacteria abundance, especially *Leptolyngbya* and *Pseudomonas*. It indicates that scalp cleaning is beneficial for itch relief. Thus, the targeted agent with microbial regulation activity in *Cutibacterium*/*Staphylococcus* might be a potential solution for scalp itch.

## Author Contributions

Conceptualization: Y.Z., M.H.Z., M.M.Z; Investigation: F.Y., X.J.L; Project administration: F.Y., X.J.L., Y.X.M; Formal analysis: X.J.L; Writing original draft: X.J.L; Writing – review and editing: M.H.Z., Y.Z.

## Ethics Statement

All the subjects were provided written informed consent willing to participate in complete test process and had been explained the procedure and the purpose of the study. The study was reviewed and approved by the Scientific and Ethical Committee at the Shanghai Jiao Tong University with No. B2020050M.

## Conflicts of Interest

The authors declare no conflicts of interest.

## Supporting information


**Data S1.** Supporting Information.

## Data Availability

The sequencing data of this study are available from the corresponding author upon reasonable request.
