# Supplementary material for: The Disturbed Microbial Niches of Itchy Scalp
Source: J Cosmet Dermatol. 2025 Jan 23;24(1):e70010. doi: 10.1111/jocd.70010 (PMC11755216; doi:10.1111/jocd.70010)
Supplement: Supplementary file 1 — Data S1. Supporting Information. [file JOCD-24-e70010-s001.docx]

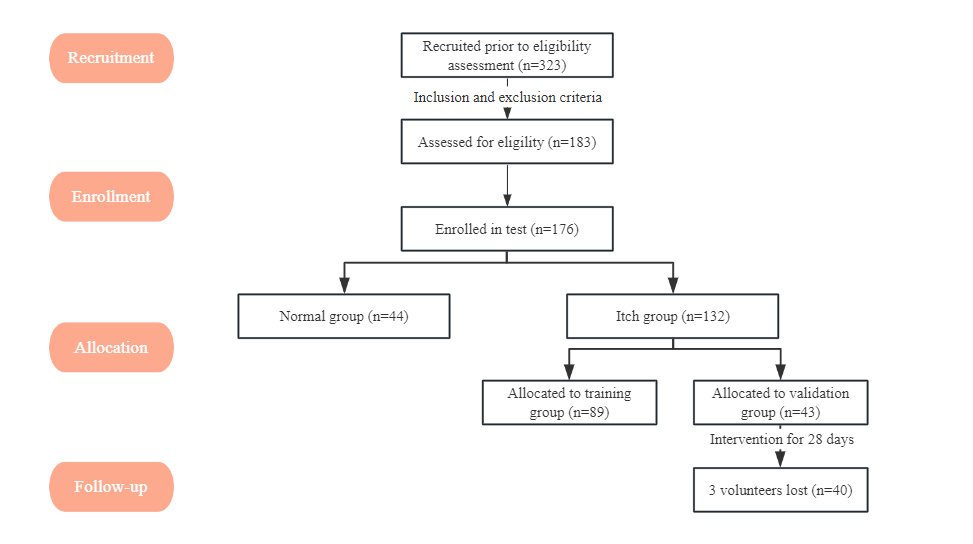


**Figure Supplementary 1. The Clinical flowchart.**

**
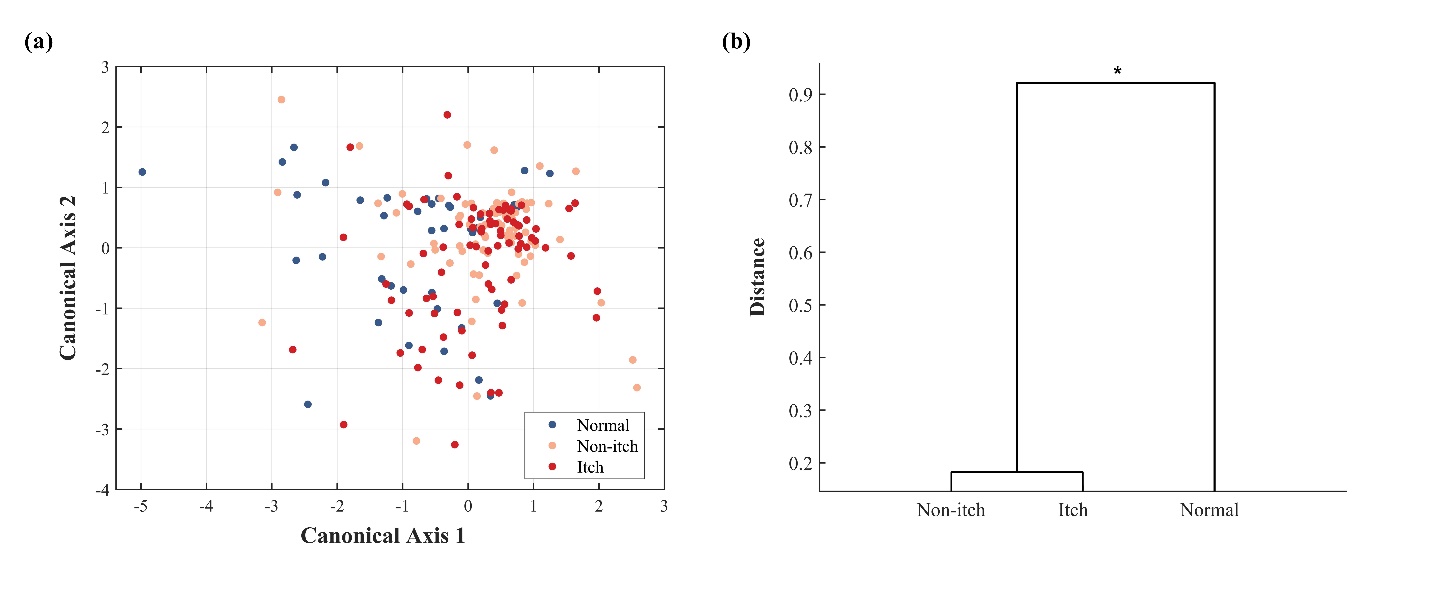
**

**Figure Supplementary 2. The microbial structure of the normal (Normal) scalp from normal subjects, itchy (Itch) and non-itchy (Non-itch) scalp sites from itchy subjects.** (a) Multivariate analysis of variance (MANOVA) plot of scalp microbiota based on the Bray Curtis distance. (b) Clustering levels among the three groups calculated with MANOVA. * P<0.05.


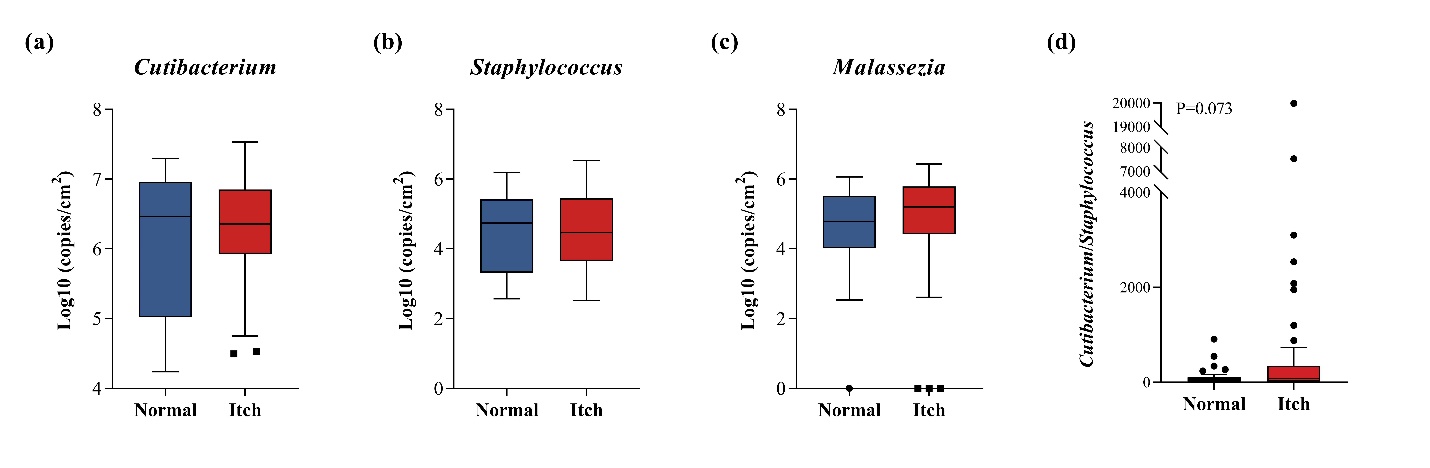


**Figure Supplementary 3. The quantification of scalp predominant bacterial genera in the normal (Normal) and itchy (Itch) group by ddPCR.** (a) *Cutibacterium*, (b) *Staphylococcus*, (c) *Malassezia*, (d) the ratio of *Cutibacterium* to *Staphylococcus*. The P value was calculated by Welch corrected t-test.

**
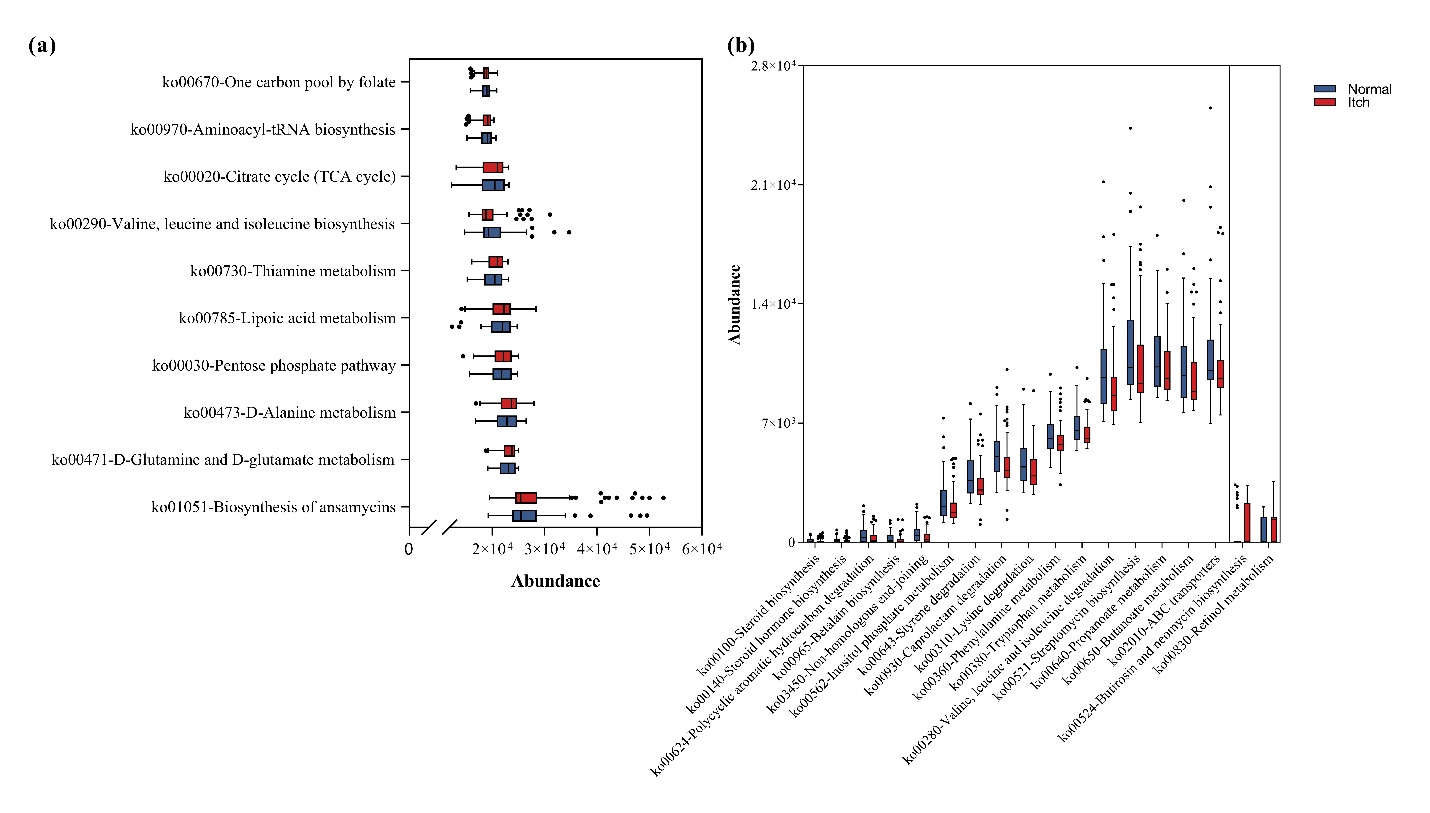
**

**Figure Supplementary 4. Functional prediction of the scalp bacterial microbiome.** (a) Distributions of the top ten abundant bacterial metabolic pathways in the normal and itchy scalp. (b) The 18 KEGG pathways relevant to scalp itch obtained by one-way ANOVA analysis. P value＜0.05

**Table Supplementary 1. The ddPCR primers for quantification of *Staphylococcus, Cutibacterium* and *Malassezia.***

| **Genus** | **Primers** | **Region** | **Primer sequence (5’-3’)** | **Amplicon size** | **Origin** |
| --- | --- | --- | --- | --- | --- |
| ***Malassezia* spp.** | Mal1F | 5.8S | TCTTTGAACGCACCTTGC | ~250bp | (Gao et al., 2010) |
|  | Mal1R | ITS2 | AHAGCAAATGACGTATCATG |  |  |
| ***Staphylococcus* spp.** | 110F | V2 | GGGTGAGTAACACGTGGATAACCT | 140bp | (Grimshaw et al., 2019) |
|  | 250R |  | GCGGCGCGGATCCATCTATAAGTG |  |  |
| ***Cutibacterium* spp.** | 591F | V4 | CGAGCGTTGTCCGGATTT | 63bp | (Clavaud C. et al., 2013) |
|  | 654R |  | CACTTCCGACGCGATCAA |  |  |

**Table Supplementary 2.** The coverage and relative abundance of 62 ASVs of *Cutibacterium* in common

| ASV ID | Coverage in Itch group (%) | Coverage in Normal group (%) | Relative abundance in Itch group (%) | Relative abundance in Normal group (%) |
| --- | --- | --- | --- | --- |
| ASV_1 | 100.00 | 97.56 | 59.66 | 53.05 |
| ASV_4 | 96.39 | 90.24 | 2.94 | 5.07 |
| ASV_43 | 18.07 | 4.88 | 1.74E-01 | 6.44E-03 |
| ASV_47 | 45.78 | 36.59 | 6.90E-02 | 1.51E-01 |
| ASV_89 | 8.43 | 12.20 | 6.86E-02 | 4.98E-03 |
| ASV_174 | 4.82 | 2.44 | 5.73E-02 | 1.46E-03 |
| ASV_175 | 22.89 | 21.95 | 1.93E-02 | 3.18E-02 |
| ASV_183 | 15.66 | 14.63 | 2.02E-02 | 3.25E-02 |
| ASV_217 | 7.23 | 9.76 | 8.96E-03 | 8.20E-03 |
| ASV_266 | 1.20 | 4.88 | 4.82E-05 | 3.71E-02 |
| ASV_327 | 19.28 | 17.07 | 9.64E-03 | 9.56E-03 |
| ASV_364 | 2.41 | 2.44 | 9.35E-03 | 9.76E-05 |
| ASV_387 | 1.20 | 2.44 | 4.82E-05 | 1.38E-02 |
| ASV_476 | 9.64 | 14.63 | 3.37E-03 | 6.93E-03 |
| ASV_512 | 6.02 | 4.88 | 1.69E-03 | 2.15E-03 |
| ASV_704 | 8.43 | 4.88 | 4.63E-03 | 2.63E-03 |
| ASV_774 | 3.61 | 7.32 | 1.30E-03 | 1.37E-03 |
| ASV_868 | 2.41 | 4.88 | 9.16E-04 | 3.90E-04 |
| ASV_892 | 7.23 | 9.76 | 1.54E-03 | 2.44E-03 |
| ASV_938 | 1.20 | 7.32 | 1.64E-03 | 2.15E-03 |
| ASV_1140 | 7.23 | 2.44 | 2.27E-03 | 1.46E-03 |
| ASV_1368 | 2.41 | 4.88 | 9.64E-05 | 2.93E-04 |
| ASV_1477 | 4.82 | 7.32 | 7.23E-04 | 3.22E-03 |
| ASV_1484 | 1.20 | 2.44 | 8.19E-04 | 1.85E-03 |
| ASV_1495 | 1.20 | 2.44 | 4.82E-05 | 9.76E-05 |
| ASV_1512 | 3.61 | 4.88 | 1.49E-03 | 3.90E-04 |
| ASV_1513 | 1.20 | 4.88 | 4.34E-04 | 3.02E-03 |
| ASV_1516 | 6.02 | 4.88 | 1.30E-03 | 1.95E-03 |
| ASV_1729 | 1.20 | 4.88 | 2.89E-04 | 1.95E-04 |
| ASV_1820 | 2.41 | 2.44 | 2.89E-04 | 1.66E-03 |
| ASV_1882 | 2.41 | 2.44 | 7.71E-04 | 9.76E-05 |
| ASV_1885 | 3.61 | 4.88 | 3.86E-04 | 1.76E-03 |
| ASV_2100 | 2.41 | 4.88 | 3.86E-04 | 1.37E-03 |
| ASV_2174 | 2.41 | 9.76 | 1.45E-04 | 1.46E-03 |
| ASV_2249 | 3.61 | 4.88 | 1.45E-04 | 1.95E-04 |
| ASV_2431 | 1.20 | 2.44 | 1.83E-03 | 9.76E-05 |
| ASV_2521 | 2.41 | 2.44 | 1.93E-04 | 8.78E-04 |
| ASV_3266 | 1.20 | 4.88 | 9.64E-05 | 8.78E-04 |
| ASV_3398 | 1.20 | 4.88 | 4.82E-05 | 1.95E-04 |
| ASV_3440 | 2.41 | 4.88 | 1.45E-04 | 7.80E-04 |
| ASV_3876 | 7.23 | 2.44 | 5.30E-04 | 9.76E-05 |
| ASV_3893 | 3.61 | 2.44 | 2.89E-04 | 9.76E-05 |
| ASV_4079 | 6.02 | 2.44 | 3.86E-04 | 1.95E-04 |
| ASV_4218 | 1.20 | 2.44 | 4.82E-05 | 1.76E-03 |
| ASV_4275 | 2.41 | 2.44 | 1.45E-04 | 1.95E-04 |
| ASV_4751 | 3.61 | 7.32 | 2.41E-04 | 3.90E-04 |
| ASV_4760 | 3.61 | 2.44 | 1.93E-04 | 1.95E-04 |
| ASV_5507 | 4.82 | 4.88 | 1.93E-04 | 5.85E-04 |
| ASV_5600 | 2.41 | 2.44 | 2.89E-04 | 9.76E-05 |
| ASV_5673 | 1.20 | 4.88 | 4.82E-05 | 3.90E-04 |
| ASV_5721 | 2.41 | 2.44 | 1.93E-04 | 1.95E-04 |
| ASV_6030 | 1.20 | 2.44 | 9.64E-05 | 1.07E-03 |
| ASV_6046 | 1.20 | 4.88 | 4.82E-05 | 3.90E-04 |
| ASV_6380 | 2.41 | 2.44 | 9.64E-05 | 1.95E-04 |
| ASV_6564 | 2.41 | 4.88 | 4.34E-04 | 1.95E-04 |
| ASV_8285 | 2.41 | 2.44 | 1.93E-04 | 1.95E-04 |
| ASV_8298 | 1.20 | 2.44 | 4.82E-05 | 2.93E-04 |
| ASV_9485 | 2.41 | 2.44 | 1.93E-04 | 9.76E-05 |
| ASV_10023 | 1.20 | 2.44 | 2.89E-04 | 9.76E-05 |
| ASV_11127 | 1.20 | 2.44 | 9.64E-05 | 1.95E-04 |
| ASV_12816 | 1.20 | 2.44 | 9.64E-05 | 9.76E-05 |
| ASV_16200 | 1.20 | 2.44 | 4.82E-05 | 9.76E-05 |

**Table Supplementary 3. Physiological indexes of subjects after intervention. (N=40)**

| **Subject Group** | **D0** | **D28** | **P value** |
| --- | --- | --- | --- |
| **VAS score** | 7.63±0.64 | 3.54±1.30 | ＜0.0001 |
| **3S scores** | 2.38±1.24 | 1.20±0.40 | ＜0.0001 |
| **ASFS** | 3.14±1.77 | 2.09±1.58 | ＜0.0001 |
| **Hydration (μS)** | 55.41±34.52 | 51.35±22.34 | 0.3755 |
| **Sebum content (a.u.)** | 335.25±155.09 | 265.30±140.33 | 0.0052 |
| **pH value** | 4.64±0.49 | 4.73±0.36 | 0.2663 |
| **Erythema index (a.u.)** | 0.09±0.07 | 0.08±0.07 | 0.6181 |

Values were shown as Mean ± SD. The P values were calculated by Mann-Whitney test.
